# Supplementary material for: Machine Learning-Based Early Warning Level Prediction for Cyanobacterial Blooms Using Environmental Variable Selection and Data Resampling
Source: Toxics. 2023 Nov 23;11(12):955. doi: 10.3390/toxics11120955 (PMC10747537; doi:10.3390/toxics11120955)
Supplement: Supplementary file 1 [file toxics-11-00955-s001.zip › toxics-2702229-supplementary.pdf]

Supplementary Material

Improving the accuracy of early warning level prediction for cyanobacterial blooms using environmental variable selection and the generation of synthetic data

Table of Contents

Contents		Page
Appendix A	Description of structure for each sampling method	2
Appendix B	Description of the RF model structure	7
Appendix C	Descriptive statistics for cyanobacteria and nutrients in the BJR	9
Appendix D	Comparison of model performance according to feature selection	10
Appendix E	Comparison of model performance according to sampling methods	11
Fig. S1	Results of the dependence test between each input variable and cyanobacteria cell density	13
Fig. S2	Comparison of confusion matrices between the RF model using original data and the optimal RF model with non-linear feature selection and CC sampling method	15
Table S1	Eutrophication standards for single parameter index in Trophic state index (Carlson, 1997)	16
Table S2(A)	Overall performance of the ANN model according to applied feature selection and sampling methods in the training step	17
Table S2(B)	Overall performance of the ANN model according to applied feature selection and sampling methods in the test step	18
Table S3(A)	Overall performance of the RF model according to applied feature selection and sampling methods in the training step	19
Table S3(B)	Overall performance of the RF model according to applied feature selection and sampling methods in the test step	20
Table S4	Optimal performance of the ANN model according to applied feature selection and sampling methods	21
Table S5	Optimal performance of the RF model according to applied feature selection and sampling methods	22
Table S6	Descriptive statistics for optimized hyperparameters obtained from the optimal model in the ANN and RF using randomly chosen training data	23

Appendix A. Description of structure for each sampling method

1) Random Oversampling (ROS)

Random Oversampling (ROS) is the simplest approach to address imbalanced data problems, where data from the minority class are randomly resampled until their size matches that of the majority class within the range of normalized values between 0 and 1. Synthetic data generated using ROS were extracted based on the data from the minority class and added to the training dataset.

2) Synthetic Minority Oversampling Technique (SMOTE)

The Synthetic Minority Oversampling Technique (SMOTE) algorithm, proposed by Chawla et al. (2002), generates synthetic data through performing random linear interpolation between the selected minority class data and its neighboring data. The specific process of generating synthetic data in SMOTE is described as follows.

1) Randomly select one of the minority class data (x_i).
2) Randomly select one of its K-nearest neighbors (x_j) from the same class (where k is a user-defined parameter, typically k = 5).
3) Calculate the distance between the selected data and its selected neighbor and multiply this distance by a random number between 0 and1 ($rand[0,1] \times (x_j - x_i)$).
4) Add the value from step (3) to the selected data ($x_i + rand[0,1] \times (x_j - x_i)$).
5) The process was iteratively performed from steps (1) to (4) until the number of minority data points equaled the number of majority data points.

Chawla, N.V., Bowyer, K.W., Hall, L.O., Kegelmeyer, W.P. (2002) Smote: synthetic minority over-sampling technique. Journal of Artificial Intelligence Research 16, 321-357.

3) Adaptive Synthetic Sampling (ADASYN)

Adaptive Synthetic Sampling (ADASYN) is an adaptive method proposed by He et al. (2008) to facilitate learning using imbalanced datasets derived from SMOTE, SMOTEBoost, and DataBoost-IM. The main concept of the ADASYN algorithm is to use a data density distribution between the minority and majority classes. This provides a basis for automatically determining the amount of synthetic data that must be generated from the small-scale data. The process of generating synthetic data using ADASYN is as follows:

1) Calculate the total number of synthetic data to be generated using the number of minority and majority data ($G = (Data_{major} - Data_{minor}) \times \beta$), where β ranges from 0 to 1, and if β is 1, generate synthetic data until it is equal to the number of the majority class.
2) Randomly select one of the minority class data points and find the K-nearest neighbors based on the Euclidean distance.
3) Calculate the ratio r_i, where r_i is the number of majority class data in the K-nearest neighbors ($r_i = number\ of\ majority\ data\ in\ cluster/K, i = 1, \dots, Data_{minor}$).
4) Calculate the density distribution ($\hat{r}_i = r_i / \sum_{i=1}^{Data_{minor}} r_i$).
5) Calculate the number of synthetic data to be generated ($g_i = \hat{r}_i \times G$).
6) Calculate the distance between the selected minority data and its selected neighbor and multiply this distance by a random number between 0 and1 ($rand[0,1] \times (x_j - x_i)$).
7) Add the value from step (6) to the selected minority data ($x_i + rand[0,1] \times (x_j - x_i)$).
8) Iteratively perform steps (6) and (7) until g_i.

He, H., Bai, Y., Garcia, E.A., Li, S. (2008) ADASYN: Adaptive synthetic sampling approach for imbalanced learning. In 2008 IEEE international joint conference on neural networks (IEEE world congress on computational intelligence, 1322-1328.

4) Cluster Centroid Undersampling (CC)

The Cluster Centroid Undersampling (CC) algorithm generates new data based on majority class data using a clustering method. The synthetic data for the majority class were generated using the cluster centroid of the K-means algorithm. The algorithm undersamples the majority class by replacing cluster centroid values of the K-means algorithm with new majority class data. However, the characteristics of the features and the amount of minority class data are preserved (Lin et al., 2017). A detailed explanation of the K-means algorithm can be found in Yadav and Sharma (2013).

Lin, W.C., Tsai, C.F., Hu, Y.H., Jhang, J.S. (2017) Clustering-based undersampling in class-imbalanced data. Information Sciences 409, 17-26.

Yadav, J., and Sharma, M. (2013) A Review of K-mean Algorithm. Int. J. Eng. Trends Technol 4(7), 2972-2976.

5) Random Undersampling (RUS)

Random undersampling (RUS) is a method of performing normalization with values from 0 to 1 for all classes based on the original data and randomly removing data from the majority class until the number of data from the majority class matches the number of data from the minority class. Random undersampling and oversampling can be performed on the data without making specific

statistical assumptions. This makes the application of this sampling method relatively straightforward compared with other sampling methods.

6) Synthetic Minority Oversampling Technique–Edited Nearest Neighbor (ENN) and Synthetic Minority Oversampling Technique–Tomek Link (Tomek)

Although oversampling can balance class distributions, it may not address other problems commonly encountered in datasets with imbalanced classes. One of these problems is that class clusters may not be well defined because of the invasion of majority class data into the space of the minority class cluster. Conversely, interpolating minority class data may expand minority class clusters and introduce synthetic data too deeply into the space of the majority class cluster, potentially leading to overfitting. To generate better-defined class clusters, Batista et al. (2004) proposed the use of undersampling methods, such as Synthetic Minority Oversampling Technique-Edited Nearest Neighbor (ENN) and Synthetic Minority Oversampling Technique–Tomek Link (Tomek) links, on the oversampled training dataset as a data cleaning method. The process of generating the dataset using SMOTE-ENN and SMOTE-Tomek involves applying SMOTE to oversample the original data and then using ENN or Tomek to perform undersampling. Consequently, a balanced dataset is generated for each well-defined class cluster. Unlike undersampling methods that involve deleting data from the majority class, SMOTE-ENN and SMOTE-Tomek are used to remove data from all classes (both majority and minority). SMOTE-ENN offers a more profound data cleaning process than SMOTE-Tomek by eliminating large amounts of data from the dataset. Wilson (1972) and Tomek (1976) provided detailed descriptions of ENN and Tomek Link methods, respectively.

Batista, G.E., Prati, R.C., & Monard, M.C. (2004) A study of the behavior of several methods for balancing machine learning training data. ACM SIGKDD explorations newsletter 6(1), 20-29.

Wilson, D.L. (1972) Asymptotic properties of nearest neighbor rules using edited data. IEEE Transactions on Systems, Man, and Cybernetics 3, 408-421.

Tomek, I. (1976) Two Modifications of CNN. IEEE Transactions on Systems, Man, and Cybernetics 6(11), 769–772.

Appendix B. Description of the RF model structure

The most significant characteristic of a random forest model is that trees are different from each other owing to randomness. This characteristic ensures that the predictions of each tree are decorrelated, leading to an improved generalization performance. Furthermore, randomization enables the forest to be effective, even with noise-containing data. Randomization was applied during the training process of each tree and random sampling of the training data was used for bagging for ensemble learning and randomized node optimization. These two methods were used simultaneously to further enhance randomization features. Bagging is a method for aggregating base learners trained on slightly different training data via bootstrapping. Bootstrapping is the process of generating a dataset with the same size as the original training data by allowing duplicates of the given training data. The process of training a random forest using bagging was conducted in three steps.

1) Generate N training datasets via the bootstrap method.
2) Train N individual trees as base classifiers.
3) Combine the trees using either averaging or majority voting to generate a single classifier.

Each tree has a variance; thus, an extremely deeply trained tree tends to overfit the training data. The bootstrapping process helps decrease the variance of trees while maintaining their biases, thereby improving the performance of the forest. A single tree is highly sensitive to noise present in the training data. However, if the trees are not correlated, the average of multiple trees is not sensitive to noise. When all trees forming the forest were trained on the same dataset, the correlation among the trees increased. Bagging trains the trees on different datasets to maintain decorrelation among the trees. In the training step, randomized node optimization is performed to

determine the optimal value of parameter θ for the node-splitting function that maximizes the training objective function. Parameter θ is defined as follows:

$$\theta = (\alpha, \beta, \gamma) \quad (1)$$

where α represents a filtering function that selects only a few features from the input factors vector. The purpose of feature selection is to address the situation in which a single feature or a small subset of features exhibits strong predictive performance for the output factor. In such cases, during the training process, these features can be redundantly selected from multiple tree nodes, resulting in correlated trees. Hence, feature selection helps mitigate the correlation between trees and improves the overall performance of the model. Parameter β represents the geometric characteristics of the splitting function, indicating the separation of the data. In general, axis-aligned hyperplanes, oblique hyperplanes, and general surfaces are used. γ represents the threshold value in the inequality of the binary test.

All trees in the forest underwent training independently. During the test step, random test data x are simultaneously inputted to all trees and reach the terminal node. The random forest prediction was obtained by averaging the predictions of all trees using the following formula:

$$p(c|x) = \frac{1}{N}\sum_{n=1}^{N} P_n(c|x) \quad (2)$$

where c represents the algal alert levels (L-0, L-1, and L-2), and $P_n(c|x)$ is the probability density function of each level in the given tree when the test data x are provided.

Appendix C. Descriptive statistics for cyanobacteria and nutrients in the BJR

N:P ratios ranged from 5.26–240.79 with an average value of 42.5. According to the N:P ratio proposed by Forsberg and Ryding (1980), a molar ratio of nitrogen to phosphorus below 10 indicates that nitrogen is the limiting nutrient for algal growth. A ratio between 10 and 17 suggests that both nutrients are limiting factors, whereas a ratio above 17 indicates that phosphorus is the limiting factor. The N:P ratio in events in all but 51 of the 345 monitoring events was above 17 (>85%); thus, phosphorus was identified as a limiting nutrient in the BJR.

For the entire monitoring period in the BJR, the concentration range and average value of Chl-a were 5.1–185.1 and 52.4 μg/L, respectively, and for phosphate, the corresponding values were 0–153 and 19.5 μg/L. From July to October, which is predominantly associated with algal bloom events corresponding to the caution and warning levels, the concentration range and average value of Chl-a were 5.3–177.7 and 50.5 μg/L, respectively. Similarly, for phosphate, during the same period, the concentration range and average values were 1–153 and 31.9 μg/L, respectively. Table S1 lists the eutrophication standards for a single parameter index in the index proposed by Carlson (1977). According to these standards, the nutritional status based on Chl-a concentration of the BJR in the entire period was classified as eutrophic (Chl-a > 7.2 μg/L) and as mesotrophic status based on phosphate (Phosphate 12–24 μg/L). However, the nutritional status from July to October was classified as eutrophic (Chl-a > 7.2 μg/L, Phosphate > 24 μg/L).

Appendix D. Comparison of model performance according to the feature selection

In terms of the comparison of predictive performance for the optimal ANN model, the model using the original data without feature selection (accuracy for the training step: 90.5%; accuracy for the test step: 90.2%) exhibited higher predictive performance compared to models using a feature selection method. For the performance of original data with the dependence test, the training accuracy was 84.0% and test accuracy was 88.2%; and for original data with MI score, the training accuracy was 84.8% and test accuracy was 88.2%. However, in the RF, the predictive performance of the model improved when feature selection methods were applied to the original dataset in both the training and test steps. In the model of the original data, the training accuracy was 88.1% and test accuracy was 88.2%. In the model of the original data with the dependence test, the training accuracy was 94.7% and test accuracy was 89.2%. In the model of the original data with an MI score, the training accuracy was 94.7% and test accuracy was 92.2%. Specifically, for L-2 events, which have the potential to expand into large-scale algal blooms, the RF model with a non-linear feature selection method provided accurate predictions for all eight L-2 events (recall of L-2 was 100%) in the test results.

Appendix E. Comparison of model performance according to sampling methods

In the overall performance comparison, the overall accuracies of the ANN and RF on the original data were relatively high at 92.6% and 92.5% in the training step. However, the overall recall, representing the accuracy of each algal alert level, was as follows: for the ANN in the training step, L-0 was 98.3%, L-1 was 66.6%, and L-2 was 82.8%; for the RF, L-0 was 98.1%, L-1 was 67.9%, and L-2 was 80.6%. As a result of applying the sampling methods for the ANN, the performance of the overall accuracy of the ROS (89.2%), SMOTE (92.0%), and ADASYN (91.9%) showed lower accuracy compared to the model trained on the original data (92.6%). However, the accuracies of CC (96.2%), RUS (96.9%), ENN (96.6%), and Tomek (92.8%) were higher in the training step. For the RF, except for CC (84.2%) and RUS (88.8%), all models (ROS: 99.2%, SMOTE: 99.7%, ADASYN: 99.6%, ENN: 99.9%, and Tomek: 99.5%) with applied sampling methods showed higher accuracy than the model trained on the original data (92.5%). When comparing the overall recall for each algal alert level across different sampling methods for the ANN and RF in the training step, it was observed that the predictive performance for L-0 remained relatively consistent, whereas the predictive performance for both L-1 and L-2 increased when using the data trained with the sampling methods compared to the original data. In other words, when the sampling methods were applied, the predictions for each algal alert level were balanced and improved. Similarly, in the test step, the overall accuracy across all types of data was generally lower than the overall accuracy of the algal alert level predictions in the training step for both the ANN and RF. However, the predictive performance for L-1 and L-2 showed an improvement in the ANN, with an average increase of 16.2% for L-1 and 6.5% for L-2, and in the RF, with an average increase of 7.1% for L-1 and 9.8% for L-2, compared with the model using the original data.

Comparing the results of the optimal model in the training step of the ANN, the accuracy for L-1 and L-2 increased in the models with the applied sampling methods compared to the models using the original data. In particular, the models with the ENN and ROS sampling methods achieved balanced predictions for each algal alert level. However, in the results of the test step, the accuracy for L-1 was lower for the ROS method at 50.0% compared to the accuracy of L-1 for the model using the original data (64.3%). Conversely, the ENN sampling method exhibited an increased accuracy of 85.7%. Furthermore, for all sampling methods except ROS, the accuracy for L-1 was higher than that for L-1 for the model using the original data in the test step. However, the accuracy of L-2 increased in models using the ROS and CC sampling methods, whereas it slightly decreased in models using the SMOTE, ADASYN, RUS, ENN, and Tomek methods. In the training step of the RF, models with all sampling methods except for CC and RUS, which are undersampling methods, showed higher accuracy compared to the models using the original data. These models provided balanced predictions for each algal alert level. In the test step, the accuracy for L-1 improved in all models, except for the model with the RUS sampling method, above the accuracy of the model using the original data for L-1. In addition, the accuracy of L-2 showed a distinct improvement in all models, except for the model with the ADASYN method applied.

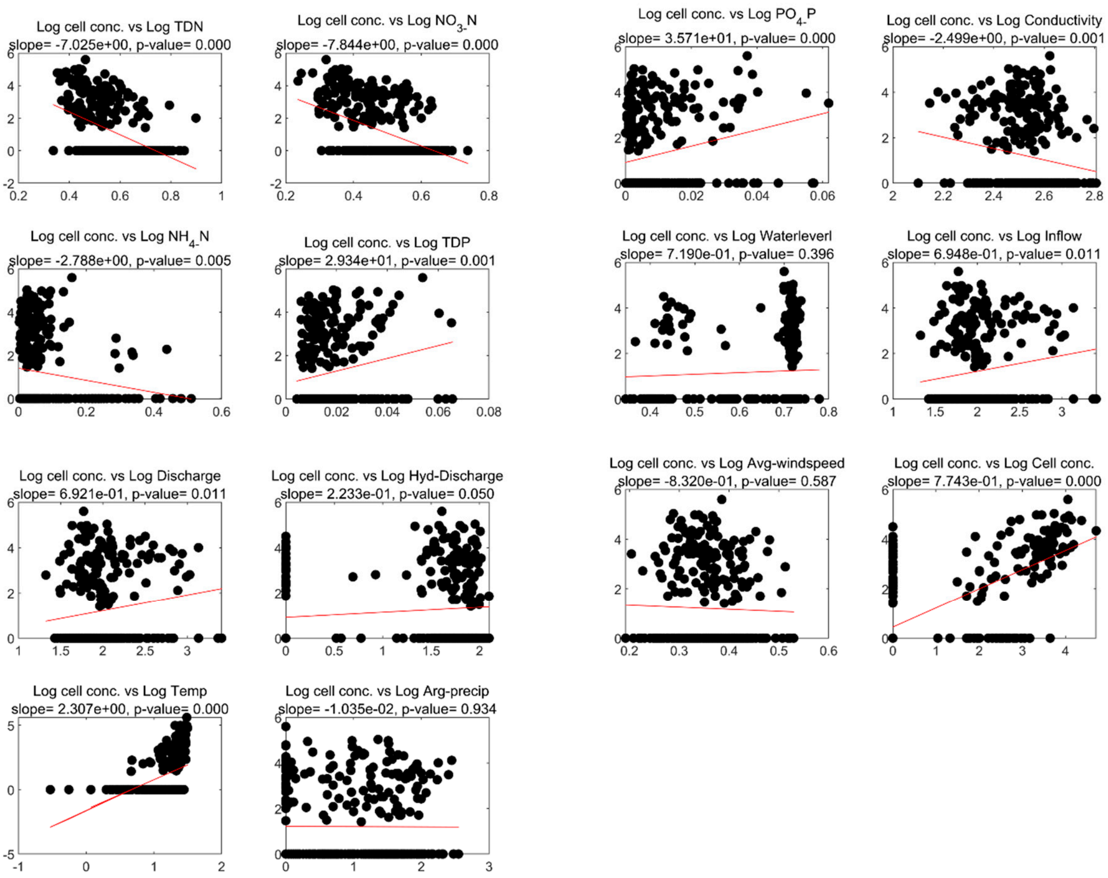

Fig. S1. Results of the dependence test between each input variable and cyanobacteria cell density.

Optimal model of RF confusion matrix

RF — Original data

Training

Observed (columns) / Predicted (rows)

	L-0	L-1	L-2	Precision
L-0	183	12	3	92.4
L-1	4	16	3	69.6
L-2	2	5	15	68.2
Recall	96.8	48.5	71.4	88.1

Test

Observed (columns) / Predicted (rows)

	L-0	L-1	L-2	Precision
L-0	79	6	3	89.8
L-1	0	7	1	87.5
L-2	1	1	4	66.7
Recall	98.8	50.0	50.0	88.2

RF — Non-linear feature selection CC sampling method

Training

Observed (columns) / Predicted (rows)

	L-0	L-1	L-2	Precision
L-0	20	4	2	76.9
L-1	1	13	0	92.9
L-2	0	4	19	82.6
Recall	95.2	61.9	90.5	82.5

Test

Observed (columns) / Predicted (rows)

	L-0	L-1	L-2	Precision
L-0	67	2	0	97.1
L-1	7	10	1	55.6
L-2	6	2	7	46.7
Recall	83.8	71.4	87.5	82.4

Fig. S2. Comparison of confusion matrices between the RF model using original data and the optimal RF model with non-linear feature selection and CC sampling method.

Table S1. Eutrophication standards for single parameter index in Trophic state index (Carlson, 1997).

Class	Total Phosphate (µg/L)	Chlorophyll-a (µg/L)	Transparence (m)
Oligotrophic	< 12	< 2.6	> 4
Mesotrophic	12 ~ 24	2.6 ~ 7.2	2 ~ 4
Eutrophic	> 24	> 7.2	< 2

Table S2(A). Overall performance of the ANN model according to applied feature selection and sampling methods in the training step.

Feature selection	Sampling method	Training (including validation)						
		Performance index						
		Accuracy	Recall			Precision		
			L-0	L-1	L-2	L-0	L-1	L-2
No feature selection	Original	92.6(±6.4)	98.3(±1.6)	66.6(±29.6)	82.8(±16.7)	94.1(±5.2)	82.5(±16.6)	87.6(±11.9)
	ROS	89.2(±8.7)	89.4(±7.8)	83.1(±14.3)	95.0(±5.7)	89.3(±8.9)	86.5(±11.0)	91.6(±7.6)
	SMOTE	92.0(±6.2)	92.3(±5.8)	87.1(±10.4)	96.7(±3.2)	90.5(±7.5)	91.0(±7.0)	94.5(±4.7)
	ADASYN	91.9(±6.9)	92.1(±6.6)	86.8(±11.5)	97.0(±3.1)	90.4(±8.4)	91.1(±7.5)	94.2(±5.2)
	CC	96.2(±6.0)	96.0(±6.3)	94.0(±10.4)	98.7(±3.4)	96.2(±6.8)	94.5(±8.5)	98.0(±4.4)
	RUS	96.9(±5.4)	97.2(±5.9)	94.8(±8.8)	98.6(±3.9)	96.5(±6.8)	96.0(±7.2)	98.2(±4.8)
	ENN	96.6(±3.0)	96.6(±3.2)	95.0(±4.7)	98.4(±2.5)	95.9(±3.9)	96.1(±3.6)	97.9(±2.4)
	Tomek	92.8(±6.5)	92.9(±6.1)	88.5(±10.7)	96.9(±3.4)	91.5(±7.9)	91.6(±7.6)	95.2(±4.7)
Linear method (Dependence test)	Original	91.7(±6.0)	98.1(±1.4)	62.7(±27.4)	79.3(±15.9)	93.4(±4.8)	80.8(±15.7)	85.0(±11.6)
	ROS	87.9(±9.9)	89.2(±8.0)	80.5(±16.6)	94.0(±7.0)	88.5(±9.5)	85.0(±12.5)	89.9(±8.8)
	SMOTE	92.1(±7.0)	93.3(±5.4)	87.4(±12.2)	95.7(±4.5)	91.5(±7.9)	91.0(±7.9)	93.8(±6.0)
	ADASYN	90.4(±7.4)	92.1(±5.7)	84.3(±12.9)	95.0(±4.9)	90.2(±8.0)	89.3(±8.3)	91.8(±6.8)
	CC	93.3(±8.7)	93.4(±10.1)	90.4(±12.9)	96.0(±6.5)	94.7(±7.7)	90.4(±12.9)	95.4(±6.6)
	RUS	92.3(±8.9)	94.6(±7.2)	88.0(±13.9)	94.1(±7.5)	93.2(±8.5)	90.2(±11.1)	93.5(±8.3)
	ENN	94.9(±4.3)	96.4(±3.1)	92.3(±6.9)	95.9(±4.4)	95.6(±4.1)	93.7(±5.4)	95.3(±4.7)
	Tomek	91.6(±7.1)	92.7(±5.5)	86.3(±12.3)	95.7(±4.6)	91.3(±7.6)	90.5(±8.1)	93.0(±6.5)
Non-linear method (MI score)	Original	91.2(±6.1)	98.2(±1.3)	59.8(±29.0)	78.5(±17.5)	93.1(±5.0)	78.8(±16.5)	84.2(±11.3)
	ROS	87.7(±9.8)	89.7(±7.6)	79.6(±17.3)	93.9(±6.8)	87.9(±9.8)	85.0(±12.2)	89.9(±9.0)
	SMOTE	91.4(±7.2)	93.0(±5.3)	85.4(±13.1)	95.9(±4.3)	90.5(±8.3)	90.6(±8.0)	93.3(±6.2)
	ADASYN	91.7(±7.5)	93.3(±5.5)	85.6(±13.8)	96.4(±3.9)	91.0(±8.6)	91.4(±7.6)	92.9(±6.7)
	CC	92.4(±8.9)	93.1(±9.6)	88.5(±14.3)	95.8(±6.3)	93.4(±8.9)	89.2(±12.5)	95.1(±7.2)
	RUS	93.7(±8.1)	96.0(±6.7)	89.7(±14.6)	95.2(±7.3)	94.6(±8.5)	92.0(±10.5)	94.7(±8.0)
	ENN	96.7(±3.8)	97.5(±3.0)	94.8(±6.3)	98.0(±3.0)	96.9(±4.0)	96.1(±4.6)	97.2(±3.8)
	Tomek	92.4(±7.2)	93.7(±5.6)	87.0(±12.8)	96.4(±4.1)	91.6(±8.3)	91.6(±7.9)	93.9(±6.2)

Table S2(B). Overall performance of the ANN model according to applied feature selection and sampling methods in the test step.

Feature selection	Sampling method	Test						
		Performance index						
		Accuracy	Recall			Precision		
			L-0	L-1	L-2	L-0	L-1	L-2
No feature selection	Original	82.4(±3.4)	93.3(±4.1)	35.6(±11.8)	55.3(±16.8)	88.9(±2.4)	55.6(±16.1)	56.3(±16.4)
	ROS	77.4(±4.3)	83.9(±5.6)	50.5(±11.3)	58.8(±19.3)	92.4(±2.8)	38.5(±8.3)	48.9(±17.1)
	SMOTE	79.4(±3.6)	86.6(±4.4)	49.6(±11.7)	59.6(±18.8)	92.3(±2.6)	42.8(±8.9)	49.8(±16.6)
	ADASYN	79.0(±3.9)	86.3(±5.2)	48.5(±12.2)	59.6(±17.0)	92.3(±2.6)	41.7(±8.3)	49.0(±15.3)
	CC	68.4(±6.0)	71.4(±7.5)	51.9(±10.5)	67.3(±16.5)	92.5(±2.4)	28.7(±7.7)	40.6(±10.5)
	RUS	64.0(±7.2)	65.5(±9.5)	53.4(±11.8)	67.9(±16.8)	93.3(±3.0)	25.8(±6.4)	37.4(±11.7)
	ENN	72.2(±5.4)	75.9(±6.4)	58.6(±12.4)	59.8(±15.8)	93.6(±2.8)	32.6(±8.0)	46.5(±14.8)
	Tomek	79.7(±3.4)	86.9(±4.2)	50.1(±12.4)	59.4(±17.8)	92.1(±3.0)	43.9(±9.4)	50.0(±14.8)
Linear method (Dependence test)	Original	83.0(±2.7)	93.7(±3.9)	38.2(±13.4)	54.9(±16.1)	89.2(±2.6)	58.0(±16.9)	60.0(±15.8)
	ROS	78.3(±4.4)	84.8(±6.0)	52.0(±11.2)	59.5(±19.3)	92.9(±3.0)	40.1(±9.1)	50.4(±15.1)
	SMOTE	81.1(±3.1)	88.3(±4.0)	52.7(±11.2)	58.4(±16.4)	92.7(±2.5)	46.5(±8.6)	52.2(±13.8)
	ADASYN	80.2(±3.7)	87.3(±4.8)	50.6(±10.0)	60.9(±18.4)	92.8(±2.6)	44.4(±8.7)	49.7(±13.8)
	CC	69.7(±5.6)	72.6(±7.3)	56.0(±11.0)	65.1(±17.0)	93.6(±2.7)	30.8(±6.9)	40.8(±11.9)
	RUS	66.0(±7.6)	68.0(±9.6)	55.1(±12.1)	65.8(±17.1)	94.0(±2.9)	27.0(±7.6)	39.5(±12.5)
	ENN	77.0(±4.8)	81.8(±5.9)	58.6(±11.5)	61.4(±18.6)	93.9(±2.5)	39.3(±9.1)	48.5(±13.6)
	Tomek	80.6(±3.4)	87.7(±4.4)	52.4(±12.2)	59.3(±18.0)	92.8(±2.6)	44.9(±8.5)	52.3(±15.5)
Non-linear method (MI score)	Original	82.2(±3.5)	93.4(±4.8)	34.9(±12.6)	53.3(±18.9)	89.0(±2.4)	54.0(±17.8)	56.7(±16.4)
	ROS	77.8(±4.4)	84.7(±6.3)	50.0(±12.5)	58.3(±19.4)	92.4(±3.0)	39.5(±9.1)	48.6(±14.7)
	SMOTE	79.8(±3.4)	87.3(±4.0)	49.9(±11.8)	58.3(±19.3)	92.3(±2.6)	43.2(±8.4)	49.5(±13.7)
	ADASYN	79.7(±3.7)	86.9(±4.3)	49.9(±12.6)	60.5(±18.6)	92.2(±2.7)	44.6(±9.6)	49.1(±14.6)
	CC	71.0(±6.5)	74.6(±8.3)	51.6(±11.4)	68.4(±17.6)	93.4(±2.6)	32.0(±9.4)	40.6(±10.8)
	RUS	64.5(±8.0)	66.5(±10.5)	54.2(±12.6)	63.1(±15.9)	92.9(±3.3)	26.3(±8.5)	39.0(±11.8)
	ENN	72.0(±5.0)	76.1(±6.3)	54.8(±11.5)	60.6(±17.4)	93.2(±2.5)	31.5(±6.6)	44.5(±14.6)
	Tomek	79.3(±3.4)	86.7(±4.1)	49.2(±12.2)	58.5(±18.2)	92.1(±3.0)	42.8(±9.0)	48.3(±13.3)

Table S3(A). Overall performance of the RF model according to applied feature selection and sampling methods in the training step.

Feature selection	Sampling method	Training (including validation)						
		Performance index						
		Accuracy	Recall			Precision		
			L-0	L-1	L-2	L-0	L-1	L-2
No feature selection	Original	92.5(±4.2)	98.1(±1.4)	67.9(±18.0)	80.6(±14.6)	94.2(±3.1)	83.1(±13.0)	88.5(±10.2)
	ROS	99.2(±6.7)	99.7(±0.8)	99.0(±10.0)	99.0(±10.0)	99.3(±6.7)	99.8(±0.6)	99.9(±0.3)
	SMOTE	99.7(±0.7)	99.4(±1.4)	99.8(±0.6)	99.9(±0.4)	99.7(±0.7)	99.5(±1.2)	99.8(±0.4)
	ADASYN	99.6(±0.8)	99.2(±1.6)	99.7(±0.7)	99.9(±0.3)	99.7(±0.8)	99.4(±1.2)	99.7(±0.5)
	CC	84.2(±8.8)	94.7(±6.7)	69.6(±20.0)	88.4(±7.5)	82.6(±12.1)	83.9(±10.9)	88.8(±7.1)
	RUS	88.8(±9.9)	93.0(±12.2)	82.0(±18.5)	91.5(±8.9)	90.8(±9.4)	86.7(±13.0)	90.6(±9.3)
	ENN	99.9(±0.3)	99.9(±0.4)	99.8(±0.5)	99.9(±0.3)	99.8(±0.4)	99.8(±0.5)	99.9(±0.3)
	Tomek	99.5(±0.9)	99.2(±1.6)	99.6(±0.9)	99.9(±0.4)	99.5(±1.0)	99.3(±1.4)	99.7(±0.5)
Linear method (Dependence test)	Original	92.4(±3.9)	98.1(±1.2)	67.5(±17.1)	80.5(±13.6)	94.2(±2.8)	82.8(±11.9)	87.2(±10.7)
	ROS	99.9(±0.3)	99.6(±0.9)	100.0(±0.2)	100.0(±0.0)	100.0(±0.2)	99.8(±0.7)	99.9(±0.3)
	SMOTE	99.5(±1.1)	99.2(±1.7)	99.6(±1.2)	99.8(±0.5)	99.6(±1.1)	99.4(±1.6)	99.7(±0.6)
	ADASYN	99.6(±0.8)	99.2(±1.6)	99.7(±0.8)	99.9(±0.2)	99.7(±0.7)	99.4(±1.3)	99.7(±0.7)
	CC	82.5(±8.9)	91.6(±8.2)	68.3(±20.7)	87.5(±7.8)	82.0(±11.6)	80.1(±11.7)	87.7(±8.0)
	RUS	85.8(±10.2)	92.5(±9.2)	75.9(±20.9)	89.0(±9.4)	87.3(±10.2)	83.6(±12.8)	88.1(±10.6)
	ENN	99.9(±0.3)	99.9(±0.4)	99.9(±0.6)	99.9(±0.3)	99.9(±0.5)	99.8(±0.5)	100.0(±0.2)
	Tomek	99.6(±0.8)	99.3(±1.4)	99.7(±0.9)	99.9(±0.3)	99.7(±0.7)	99.5(±1.2)	99.7(±0.7)
Non-linear method (MI score)	Original	93.5(±4.4)	98.3(±1.6)	72.1(±18.5)	83.1(±14.3)	95.0(±3.2)	85.5(±12.7)	89.5(±10.9)
	ROS	99.9(±0.3)	99.8(±0.7)	100.0(±0.1)	100.0(±0.0)	100.0(±0.1)	99.9(±0.5)	99.9(±0.3)
	SMOTE	99.3(±1.2)	98.9(±1.9)	99.4(±1.4)	99.8(±0.5)	99.4(±1.3)	99.1(±1.8)	99.6(±0.7)
	ADASYN	99.7(±0.8)	99.3(±1.5)	99.7(±0.9)	99.9(±0.5)	99.8(±0.7)	99.6(±1.2)	99.7(±0.7)
	CC	83.7(±7.0)	93.0(±7.5)	70.8(±15.5)	87.1(±6.9)	82.8(±10.3)	82.4(±9.5)	87.9(±5.6)
	RUS	86.4(±10.3)	93.5(±7.6)	75.0(±22.3)	90.8(±8.7)	87.1(±10.7)	85.4(±12.9)	88.4(±10.6)
	ENN	99.8(±0.5)	99.8(±0.5)	99.7(±1.0)	99.9(±0.3)	99.8(±0.7)	99.8(±0.6)	99.9(±0.4)
	Tomek	99.6(±0.8)	99.3(±1.4)	99.6(±1.0)	99.9(±0.2)	99.7(±0.8)	99.5(±1.0)	99.7(±0.7)

Table S3(B). Overall performance of the RF model according to applied feature selection and sampling methods in the test step.

Feature selection	Sampling method	Test						
		Performance index						
		Accuracy	Recall			Precision		
			L-0	L-1	L-2	L-0	L-1	L-2
No feature selection	Original	84.4(±2.4)	95.8(±2.2)	36.4(±11.4)	54.4(±15.7)	89.7(±2.1)	53.8(±14.6)	67.5(±17.3)
	ROS	83.2(±2.9)	93.8(±3.3)	37.3(±13.0)	56.6(±17.4)	89.9(±2.7)	50.4(±14.6)	61.6(±16.2)
	SMOTE	82.3(±2.8)	91.3(±3.3)	42.3(±12.4)	63.3(±16.7)	91.9(±2.2)	44.3(±10.7)	59.3(±14.5)
	ADASYN	82.2(±3.3)	91.1(±3.5)	42.6(±13.6)	62.9(±16.7)	92.0(±2.1)	44.0(±12.2)	59.4(±15.1)
	CC	73.0(±7.2)	78.4(±9.5)	41.7(±14.0)	73.1(±13.6)	92.5(±2.6)	38.7(±16.1)	38.2(±12.0)
	RUS	71.8(±9.0)	76.7(±12.4)	46.6(±16.9)	66.9(±17.5)	94.0(±2.7)	29.8(±10.5)	43.5(±13.1)
	ENN	77.4(±4.4)	83.6(±5.8)	50.4(±14.1)	62.9(±19.3)	93.7(±2.5)	34.3(±7.3)	55.5(±14.3)
	Tomek	82.5(±2.9)	91.1(±3.3)	43.6(±12.5)	63.9(±17.2)	91.8(±2.2)	44.6(±10.0)	61.4(±15.1)
Linear method (Dependence test)	Original	85.2(±2.5)	96.0(±2.5)	40.1(±11.1)	56.9(±16.4)	90.2(±2.2)	60.2(±14.8)	66.8(±14.5)
	ROS	83.8(±2.9)	94.2(±2.9)	40.7(±13.6)	55.0(±16.0)	90.7(±2.4)	50.8(±13.9)	62.2(±15.9)
	SMOTE	82.8(±3.3)	91.5(±3.2)	44.7(±13.5)	62.5(±17.6)	92.3(±2.4)	45.7(±11.3)	60.0(±14.3)
	ADASYN	82.6(±2.8)	91.1(±2.9)	45.1(±12.7)	63.3(±18.4)	92.4(±2.4)	45.4(±9.9)	58.9(±14.8)
	CC	71.9(±8.0)	77.1(±10.5)	41.6(±15.8)	73.1(±14.8)	92.3(±2.7)	35.8(±16.6)	40.3(±13.7)
	RUS	71.8(±8.2)	76.7(±11.1)	45.3(±15.7)	68.9(±16.6)	93.8(±2.7)	30.1(±10.7)	43.6(±11.7)
	ENN	80.0(±3.7)	86.7(±4.3)	50.1(±15.1)	65.1(±17.6)	93.5(±2.5)	39.5(±8.7)	56.0(±15.1)
	Tomek	83.0(±3.0)	91.6(±3.0)	45.2(±14.0)	63.1(±18.1)	92.2(±2.2)	46.8(±10.9)	59.4(±14.1)
Non-linear method (MI score)	Original	84.5(±2.8)	95.3(±2.8)	38.3(±12.8)	56.8(±17.4)	89.9(±2.3)	55.6(±14.4)	65.4(±15.3)
	ROS	83.0(±3.1)	93.4(±3.3)	39.7(±13.6)	54.5(±17.1)	90.6(±2.4)	49.3(±14.0)	57.9(±16.1)
	SMOTE	81.7(±2.9)	90.6(±3.1)	42.9(±13.1)	60.9(±17.5)	91.9(±2.3)	42.0(±9.8)	59.1(±15.2)
	ADASYN	81.8(±2.8)	90.5(±3.3)	43.2(±12.4)	61.9(±16.5)	92.1(±2.1)	43.0(±10.2)	57.4(±15.0)
	CC	72.9(±7.8)	78.1(±10.3)	41.9(±15.1)	75.8(±12.7)	92.6(±2.5)	39.7(±16.2)	37.3(±11.5)
	RUS	73.3(±6.8)	79.2(±8.8)	41.5(±16.3)	69.6(±16.6)	93.1(±2.8)	29.9(±10.9)	44.7(±12.7)
	ENN	78.1(±4.0)	84.6(±5.4)	48.6(±13.4)	64.9(±16.2)	93.3(±2.6)	35.1(±7.7)	56.3(±14.4)

Tomek	82.0(±2.7)	90.7(±3.2)	43.9(±13.1)	62.0(±17.4)	92.0(±2.2)	43.5(±8.7)	58.1(±13.9)

Table S4. Optimal performance of the ANN model according to applied feature selection and sampling methods.

Feature selection	Sampling method	Training (including validation)							Test						
		Performance index							Performance index						
		Accuracy	Recall			Precision			Accuracy	Recall			Precision		
			L-0	L-1	L-2	L-0	L-1	L-2		L-0	L-1	L-2	L-0	L-1	L-2
No feature selection	Original	90.5	96.3	63.6	81.0	93.3	75.0	85.0	90.2	96.2	64.3	75.0	92.8	75.0	85.7
	ROS	96.1	91.5	96.8	100	96.6	94.3	97.4	82.4	87.5	50.0	87.5	92.1	63.6	46.7
	SMOTE	86.4	90.5	75.1	93.7	81.4	87.7	90.8	85.3	90.0	78.6	50.0	94.7	57.9	57.1
	ADASYN	83.7	85.7	73.8	92.0	80.2	82.3	88.7	83.3	87.5	78.6	50.0	95.9	47.8	66.7
	CC	87.3	95.2	81.0	85.7	100	81.0	81.8	79.4	81.2	64.3	87.5	94.2	40.9	63.6
	RUS	90.5	100	85.7	85.7	95.5	85.7	90.0	67.6	67.5	78.6	50.0	93.1	28.9	66.7
	ENN	95.5	95.5	94.3	97.0	93.9	95.9	97.0	81.4	83.8	85.7	50.0	95.7	50.0	50.0
	Tomek	87.0	88.8	78.6	93.7	84.7	83.1	93.2	82.4	85.0	78.6	62.5	97.1	68.8	31.2
Linear method (Dependence test)	Original	84.0	96.8	27.3	57.1	88.0	50.0	70.6	88.2	98.8	42.9	62.5	89.8	85.7	71.4
	ROS	94.2	90.5	92.1	100	91.9	91.6	99.0	87.3	93.8	64.3	62.5	93.8	69.2	55.6
	SMOTE	93.5	93.1	89.4	97.9	89.8	93.4	97.4	83.3	88.8	71.4	50.0	92.2	58.8	50.0
	ADASYN	82.6	88.9	72.3	87.0	83.6	77.9	86.0	84.3	87.5	71.4	75.0	93.3	58.8	60.0
	CC	93.7	90.5	90.5	100	90.5	90.5	100	70.6	72.5	78.6	37.5	95.1	36.7	27.3
	RUS	79.4	85.7	76.2	76.2	90.0	72.7	76.2	80.4	81.2	78.6	75.0	97.0	45.8	54.5
	ENN	90.1	94.8	86.9	88.2	95.6	85.3	89.1	87.3	92.5	64.3	75.0	96.1	69.2	50.0
	Tomek	82.8	90.3	66.3	91.5	82.3	80.3	85.1	85.3	90.0	71.4	62.5	91.1	62.5	71.4
Non-linear method (MI score)	Original	84.8	96.3	24.2	76.2	87.5	53.3	80.0	88.2	98.8	50.0	50.0	88.8	87.5	80.0
	ROS	77.6	86.8	65.1	81.0	80.8	69.5	81.8	83.3	87.5	64.3	75.0	93.3	56.2	54.5
	SMOTE	80.2	91.5	58.7	90.5	75.2	81.0	85.5	85.3	87.5	78.6	75.0	97.2	52.4	66.7
	ADASYN	91.5	91.0	88.3	95.3	95.6	90.6	88.9	89.2	93.8	64.3	87.5	94.9	75.0	63.6
	CC	77.8	85.7	61.9	85.7	78.3	72.2	81.8	70.6	70.0	78.6	62.5	100	32.4	41.7
	RUS	82.5	85.7	76.2	85.7	85.7	72.7	90.0	80.4	81.2	71.4	87.5	98.5	58.8	36.8
	ENN	90.9	95.6	86.2	90.5	93.1	87.9	91.5	80.4	82.5	71.4	75.0	95.7	50.0	46.2
	Tomek	83.3	87.5	75.0	87.3	87.0	77.5	85.1	90.2	97.5	57.1	75.0	94.0	72.7	75.0

Table S5. Optimal performance of the RF model according to applied feature selection and sampling methods.

Feature selection	Sampling method	Training (including validation)							Test						
		Performance index							Performance index						
		Accuracy	Recall			Precision			Accuracy	Recall			Precision		
			L-0	L-1	L-2	L-0	L-1	L-2		L-0	L-1	L-2	L-0	L-1	L-2
No feature selection	Original	88.1	96.8	48.5	71.4	92.4	69.6	68.2	88.2	98.8	50.0	50.0	89.8	87.5	66.7
	ROS	99.5	100	100	99.8	100	100	99.5	85.3	93.8	50.0	62.5	88.2	63.6	83.3
	SMOTE	98.4	97.9	98.4	98.9	97.4	99.5	98.4	85.3	90.0	50.0	100.0	94.7	58.3	57.1
	ADASYN	99.3	97.9	100	100	100	98.5	99.5	83.3	90.0	71.4	37.5	91.1	58.8	50.0
	CC	73.0	95.2	42.9	81.0	66.7	75.0	81.0	82.4	87.5	50.0	87.5	93.3	87.5	36.8
	RUS	77.8	100	38.1	95.2	75.0	100	74.1	86.3	96.2	35.7	75.0	90.6	83.3	54.5
	ENN	99.4	100	99.1	99.0	99.1	99.1	100	86.3	85.0	85.7	100	98.6	54.5	72.7
	Tomek	100	99.5	100	99.8	99.5	100	100	88.2	95.0	57.1	75.0	92.7	72.7	66.7
Linear method (Dependence test)	Original	94.7	98.9	75.8	85.7	94.9	89.3	100	89.2	98.8	50.0	62.5	89.8	100	71.4
	ROS	99.6	98.9	100	100	100	100	99.0	91.2	96.2	71.4	75.0	93.9	76.9	85.7
	SMOTE	99.6	98.9	100	100	100	99.0	100	84.3	87.5	78.6	62.5	94.6	52.4	71.4
	ADASYN	99.7	99.5	99.5	100	99.5	100	99.5	88.2	92.5	78.6	62.5	96.1	57.9	83.3
	CC	71.4	90.5	57.1	66.7	70.4	66.7	77.8	81.4	86.2	85.7	25.0	95.8	46.2	50.0
	RUS	77.8	100	57.1	76.2	75.0	70.6	88.9	83.3	87.5	85.7	37.5	100	46.2	50.0
	ENN	98.2	98.5	96.3	100	97.7	98.1	98.9	83.3	83.8	85.7	75.0	97.1	46.2	85.7
	Tomek	97.7	96.2	97.3	99.5	97.8	95.8	99.5	84.3	90.0	78.6	37.5	92.3	57.9	60.0
Non-linear method (MI score)	Original	94.7	97.9	81.8	85.7	95.9	87.1	94.7	92.2	100	42.9	100	92.0	100	88.9
	ROS	99.6	98.9	100	100	100	99.0	100	87.3	95.0	64.3	50.0	93.8	64.3	57.1
	SMOTE	96.3	94.7	96.3	97.9	96.8	94.8	97.4	86.3	91.2	78.6	50.0	94.8	55.0	80.0
	ADASYN	98.1	94.2	100	100	100	95.6	98.9	85.3	91.2	64.3	62.5	94.8	52.9	62.5
	CC	82.5	95.2	61.9	90.5	76.9	92.9	82.6	82.4	83.8	71.4	87.5	97.1	55.6	46.7
	RUS	90.5	95.2	81.0	95.2	90.9	89.5	90.9	84.3	87.5	71.4	75.0	97.2	52.6	54.5
	ENN	99.1	98.2	99.1	100	100	98.3	99.0	79.4	82.5	78.6	50.0	97.1	39.3	66.7
	Tomek	95.6	94.1	93.1	99.5	96.2	95.6	94.9	88.2	91.2	78.6	75.0	100	55.0	66.7

Table S6. Descriptive statistics for optimized hyperparameters obtained from optimal model in the ANN and RF using randomly chosen training data.

Model	Hyperparameter	
ANN	Activation function	Linear
	Number of hidden neurons	24
RF	Ensemble aggregation method	Adaptive boosting
	Number of ensembles learning cycles	25
	Learning rate for shrinkage	0.087
	Minimum leaf size	4
	Maximum number of decision splits	14
	Number of predictors to select at random for each split	13
